# Exacerbation of Pre-existing Neurological Symptoms With COVID-19 in Patients With Chronic Neurological Diseases: An Updated Systematic Review

**DOI:** 10.7759/cureus.29297

**Published:** 2022-09-18

**Authors:** Md Sakibuzzaman, Anid Hassan, Samira Hayee, Fariah Asha Haque, Sumaita Sadida Bushra, Maisha Maliha, Maksuda Khan Tania, Anahita Sadat, Fahima Akter, Tanusree Mazumder, Joyeta Razzaque, Progga Kapuria, Ishra Jalal, Prince Shah-Riar

**Affiliations:** 1 Department of Neurology, University of Toledo, Toledo, USA; 2 Department of Internal Medicine, Holy Family Red Crescent Medical College Hospital, Dhaka, BGD; 3 Department of Internal Medicine, Chittagong Medical College and Hospital, Chittagong, BGD; 4 Department of Internal Medicine, Jawaharlal Nehru Medical College (JNMC) and Acharya Vinoba Bhave Rural (AVBR) Hospital, Wardha, IND; 5 Department of Internal Medicine, Jahurul Islam Medical College and Hospital, Kishoreganj, BGD; 6 Department of Internal Medicine, Dhaka Medical College and Hospital, Dhaka, BGD; 7 Department of Internal Medicine, Ibrahim Medical College and Birdem General Hospital, Dhaka, BGD; 8 Department of Internal Medicine, Zainul Haque Sikder Women's Medical College and Hospital, Dhaka, BGD; 9 Department of Internal Medicine, Khulna Medical College and Hospital, Khulna, BGD; 10 Department of Gastroenterology, Ibn Sina Medical College, Dhaka, BGD

**Keywords:** alzheimer's disease, epilepsy, parkinson's disease, stroke, pre-existing neurological disorders, covid-19

## Abstract

The neurotropism of the severe acute respiratory syndrome coronavirus 2 (SARS-CoV-2) can potentially explain the worsening of symptoms in patients with a history of neurological conditions such as stroke, Parkinson's disease, Alzheimer's, and epilepsy. Several studies have reported that these pre-existing conditions may worsen with a higher frequency of flare-ups, thus resulting in a more significant risk of patient mortality. In this review, we sought to provide an overview of the relationship between pre-existing neurological disorders and COVID-19, focusing on whether the initial infection directly influenced the severity of symptoms. We systematically searched the electronic database PubMed (MEDLINE) and used specific keywords related to our aims from January 2020 to July 2022. All articles published on COVID-19 with keywords pertaining to pre-existing neurological diseases were retrieved and subsequently analyzed. After independent review, the data from 107 articles were selected and evaluated. After analyzing the data from selected articles reviewing the effect of COVID-19 on neurological conditions, we have documented the relationship between said pre-existing neurological diseases, showing an increased risk of hospitalization, admission length, worsening of symptoms, and even mortality in COVID-19 patients.

## Introduction and background

The 2019 severe acute respiratory syndrome coronavirus 2 (SARS-CoV-2) pandemic caused a profound impact, infecting three million lives and causing a death toll of almost 600,000 people alone in the United States. The crisis led to enormous patient overload in health care facilities worldwide, with symptoms often leading to profound multiorgan failure and high mortality rates. One of the systems the SARS-CoV-2 frequently infiltrated was its access within the central nervous system (CNS), and the neurotropism of COVID-19 can explain the exacerbation of neurological symptoms in patients with a history of stroke, Parkinson's disease (PD), Alzheimer's disease, multiple sclerosis, and epilepsy [1].

The high viral spread of the SARS-CoV-2 raised successive sequela of long-term concerns particular to those with underlying severe neuro-medical illnesses, leaving patients with severe additional impairments to their functional disabilities [2]. These CNS complications are caused via the SARS-CoV-2’s inflammatory responses within the immune system, with additional consequences resulting in patients with symptoms of ischemic or hemorrhagic stroke having a higher risk of mortality than other hospitalized patients [3]. Patients exhibiting pre-existing brain pathologies- such as dementia and Alzheimer's- experienced exacerbating symptoms that caused the further cognitive functional decline. Those with PD and multiple sclerosis also experienced heightened mobility issues when exposed to SARS-CoV-2 [4]. In addition to the emotional and social isolation resulting from the pandemic, most patients affected by pre-existing degenerative motor disorders were unable to get proper in-person evaluations and thus had a greater risk of their comorbid conditions developing into severe pneumonia and being admitted to critical care [5]. Patients with uncontrolled epilepsy were also more likely to experience more significant health impacts from COVID-19, as medically induced sedation and ventilations would present a risk of emerging seizures during treatment and would be difficult to suppress due to epilepsy or severe immune dysfunction being refractory to possible medications [6]. Therefore, the current study aimed to search whether the patients with pre-existing neurological disorders had a significant exacerbation of their symptoms and whether their severity was due to the initial infection.

## Review

Methodology

Following Preferred Reporting Items for Systematic Reviews and Meta-Analyses (PRISMA) guidelines [7], a systematic review was conducted based on the articles published from January 1, 2020 to July 31, 2022. This review was not officially registered. For data collection, we performed a comprehensive search strategy online using MEDLINE (accessed from Pubmed.) We searched the database using search terms including but not limited to “pre-existing neurological disorders and COVID-19”, “exacerbation of neurological disorders,” “new neurological symptoms,” “COVID-19”, “Corona Virus and Neurological disorders,” “Parkinson’s Disease,” “Stroke,” “Multiple Sclerosis,” “Alzheimer’s Disease,” “Epilepsy” “Dementia,” “pre-existing and nervous system disease,” “Neurological disorders and COVID.”

We included the studies that met the following criteria: 1) All the studies have PICO (Patient or problem, Intervention or exposure, Comparison or control) framework, 2) Original articles such as systematic reviews and meta-analyses, cross-sectional studies, case-control studies, longitudinal studies, case reports, case series were included, 3) aged over 18 years, 4) patients diagnosed with COVID-19 according to World Health Organization (WHO) standards reporting exacerbation of pre-existing neurological diseases or newly developed neurological symptoms and 5) Articles reporting patients with pre-existing neurological diseases and COVID-19. We excluded pediatric cases (age<18 years), studies written in foreign languages, non-neurological disorders, neuropsychiatric disorders like schizophrenia, mood disorders, pre-clinical trials, studies on vaccine or drug effects, and studies with different outcomes from the final review.

The severity of COVID-19 was defined as any patient who needed admission to the hospital or Intensive Care Unit (ICU) with severe respiratory distress, low oxygen saturation, or any other critical complications. A total of 1,273 articles were retrieved relevant to our initial keywords from the online database. Eight independent reviewers then screened the titles and abstracts and excluded the duplicate 1,118 non-relevant studies. In the end, a full-text screening of the remaining 162 studies was completed to check for eligibility. Subsequently, the studies were subcategorized according to the aforementioned neurological conditions. Additional four reviewers resolved any controversies during the screening steps.

**Figure 1:**
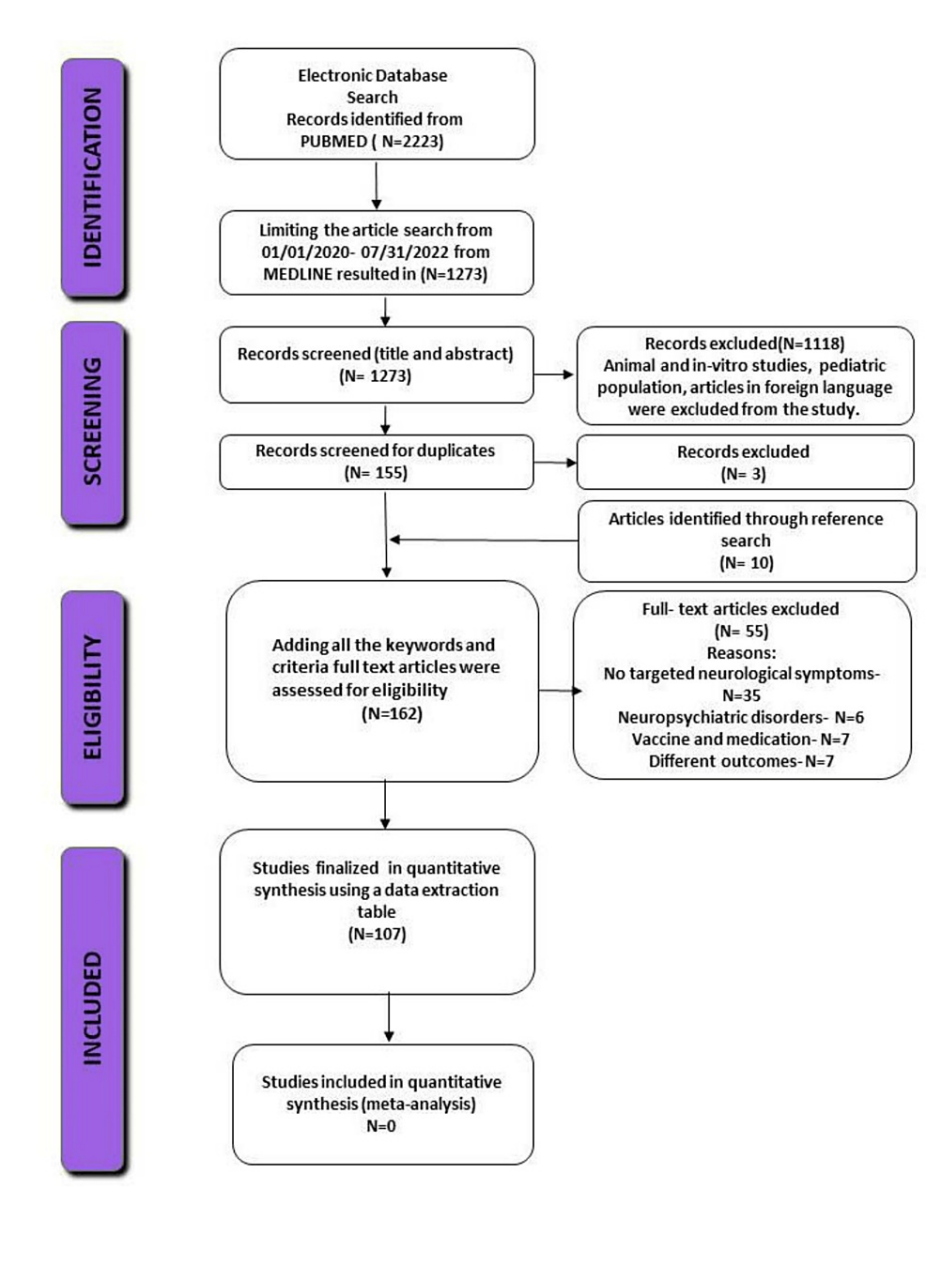
PRISMA flow diagram of the included studies

Finally, a total of (N=107) studies were selected as eligible for review (Figure 1). All the articles went through extensive deliberations, scanning the relevant information and paraphrasing the essential points for analysis in google spreadsheets for four months. Any discrepancies were solved among the authors by thorough discussion.

Our systematic review had some limitations. Firstly, the studies were searched only in PubMed. However, we limited it to Pubmed to ensure good quality and avoid predatory articles. Furthermore, some confounders may have influenced the correlation between COVID-19 and worsening neurological disease symptoms. Finally, there might be some nonuniformity between the included research articles. For example, study size, neurological complications, study designs, and data collection varied among the included studies; hence, the findings must be interpreted cautiously (Tables 1, 2).

**Table 1:** Neurological symptoms and severity in patients with pre-existing chronic neurological diseases

Name of disease	New symptoms after COVID-19	Hospitalization due to COVID-19	COVID-19 related mortality
Stroke	Anosmia, fatigue, dizziness, headache, and myalgia; Dizziness, Headache, Confusion, Altered mental status, Facial droop, Slurred speech, weakness, and Paralysis; Difficulties in memory, poor concentration, visual disturbance, decreased consciousness levels, seizures, delirium, involuntary movements, and ataxia	Duration of hospital stay was generally increased.	COVID-19 patients with stroke had higher chances of worse outcomes and more significant mortality than those without.
Parkinson’s disease (PD)	Exaggeration of motor symptoms was more common than non-motor symptoms	Increased hospitalization and ICU admission rate among known Parkinson’s patients	Higher mortality rate compared to patients with no neurologic disorders
Delirium	Not related to developing new symptoms.	Patients previously diagnosed with dementia are more likely to develop delirium and less likely to exhibit some other COVID-19 symptoms on presentation.	Delirium associated with COVID-19 has been associated with increased mortality.
Alzheimer’s disease (AD)	Neuro-degeneration, Cognitive decline memory deterioration, behavioral changes, orientation abilities, reduced levels of independence, functional decline, and new or deteriorating behavioral disturbances, including aggression, apathy, and depression	People with Alzheimer's Disease are at high risk of contracting COVID-19 and develop worsening outcome	Increased risk of mortality
Multiple sclerosis (MS)	Multiple Sclerosis exacerbation or recrudescence or additional signs not typical of MS	Increased in Progressive MS subtype or presence of other comorbidities	Not increased
Epilepsy	Headache, Myalgia, Delirium, Parageusia, Anosmia, Limb weakness, Distorted mental status after withdrawal of sedatives,	Not increased	Increased risk of mortality and sudden unexpected death.

**Table 2:** Summary of patients with neurological disorders and COVID-19 NA = Not available, SD = Standard Deviation

Author (Reference)	Study Design	Age (mean ± SD)	Male (%)	Total Number of patients with neurological disorders	Types of neurological disorder	Key exacerbating symptoms or severe symptoms	Number of patients with preexisting neurological disorders having severe COVID symptoms	Death or Mortality rate (%)
Asadi‐Pooya et al. [8]	Case control study	52±23	53.2	1086	Epilepsy, cerebrovascular accident, Alzheimer's disease, Parkinson's disease, Multiple sclerosis, Unspecified	Seizure	NA	Case fatality rate 8.5%
Brown et al. [9]	Cross sectional study	65	73%	5429	Parkinson's disease	Motor and Non-motor symptoms (mood, cognition, sleep, autonomic)	NA	NA
Drabik et al. [10]	Cohort study	64	45.28	349	Stroke, Dementia, Parkinson's disease, Epilepsy, CNS tumor, Traumatic brain injury	Headache, dizziness, dementia, decreased consciousness, stroke, seizure, ataxia	NA	9.46
El‐Qushayri et al. [11]	Systematic review and meta-analysis	NA	NA	8649	Parkinson's disease	NA	Poor in-hospital outcomes of COVID-19 [OR 2.64 (95% CI 1.75–3.99), p < 0.00001	25.10%
Eskandar et al. [12]	Case control study	65	NA	581	Stroke, Seizure	Altered mentation, Stroke, Seizure, Neuro covid 19 complexes	NA	58.70%
Fan et al. [13]	Cross sectional study	NA	NA	(1836+3060)=4864	Multiple Sclerosis, Neuromyelitis Optica Spectrum disorders	NA	2	NA
Flores-Silva, et al. [14]	Cross sectional study	53.2 ±13.7	65	163	Epilepsy, Ischemic stroke, Multiple sclerosis, and Parkinson’s disease	Seizure, Stroke, Delirium, Myopathy, Headache	NA	19.60%
Garjani et al. [15]	Prospective cohort study	49 (<4wks) 50 (≥4wks) 51 (>12 wks.)	25.9 (<4wks) 17.6 (≥4wks)15.5 (>12wks)	7977	Multiple sclerosis	Change in smell or taste, headache, fever, New muscle pain ,and New or worse fatigue, Upper respiratory tract, lower respiratory tract and Gastrointestinal tract symptoms	1096	NA
Harb et al. [16]	Cohort study	84 (for patients with dementia)	43.1	116	Dementia	Delirium, Dyspnea	50/116 (developed ARDS)	50
Hu et al. [17]	Cohort study	74.2 ±5.84	53	2617	Neurodegenerative disease	NA	379	7.5
Huber et al. [18]	Prospective cohort study	Median decade 76-85years	59	330	Parkinson’s disease, Dementia	Dry cough, Dyspnea, Fever, Delirium, Headache, Taste disorder	NA	32.5 (Parkinson's disease) 32.1 (Dementia)
Kubota et al. [19]	Systematic review	NA	NA	232	Dementia, Parkinson’s disease, Epilepsy, and Unspecified neurological disorders	Dementia, Parkinson’s disease, Multiple sclerosis, Epilepsy	478	NA
Kuroda et al. [20]	Review article	NA	NA	NA	Epilepsy	Seizure	NA	NA
Louapre et al. [21]	Cohort study	44.6±12.8	28.2	347	Multiple sclerosis	ARDS	73	3.46
Nejad et al. [22]	Cross-sectional study	Different age group was compared	57.2	891	Stroke, Seizure	Headache, sleeping problem, anosmia, dizziness, hypogeusia, memory issues, seizure	NA	NA
Numbers and Brodaty [23]	Review article	NA	NA	NA	Alzheimer’s disease, Dementia	Severe virus related outcome including death	NA	Mortality increased
Parrotta et al. [24]	Cross-sectional	44.9 ± 15.2	38.2	72	Multiple sclerosis	Relapsing of neurological symptoms	8	8.33
Santos‐García et al. [25]	Cross- sectional study	63.5±12.5	47	NA	Parkinson's disease	Tremor, rigidity, fluctuation of motor symptoms, etc.	NA	NA
Yassin et al. [26]	Systematic review and meta analysis	50.3	53	NA	NA	Myalgia, taste impairment, smell impairment, headache, dizziness, encephalopathy, CVD.	NA	NA
Zhang et al. [27]	Case series	70( for stroke patients)	67	49	Stroke	Sepsis, ARDS, Acute kidney injury	NA	45

Discussion

*Neurotropism of COVID-19 and Its Role in Patients With Pre-Existing Neurological Conditions*****

**Figure 2:**
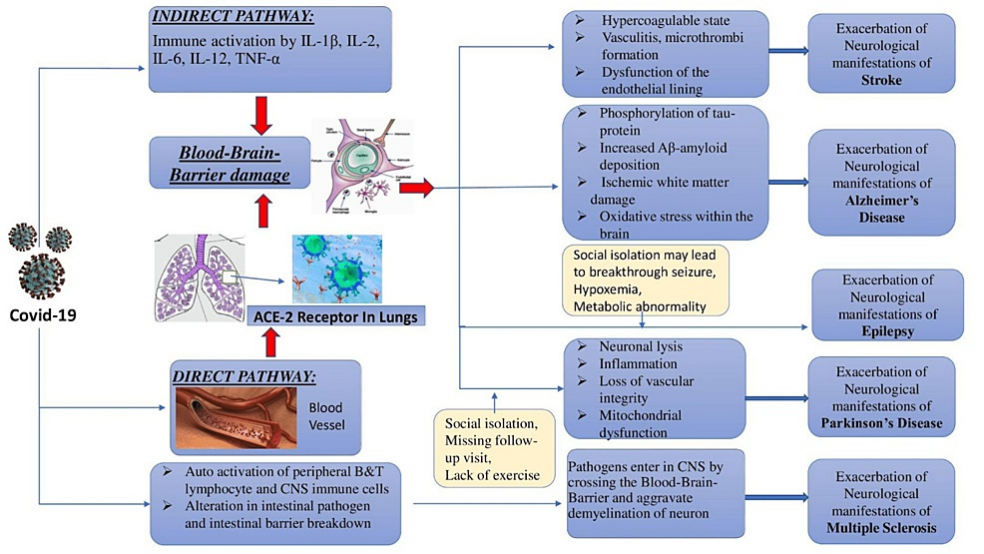
Mechanism of aggravation of neurological manifestations due to COVID-19 This figure is a self-illustration by the authors.

As a consequence of COVID-19, several cases presented with various neurological complications [28]. These patients have a worse neurological outcome than the general population's neurological symptom exacerbation. Therefore, our study evaluates the exacerbation of neurological manifestations and the intensity of COVID-19 in patients with pre-existing neurological disorders (Figure 2) [29].

Pathophysiology of COVID-19-Related Stroke

Three main mechanisms responsible for ischemic strokes in COVID-19 include a hypercoagulable state, vasculitis, and cytokine storm [30].

Hypercoagulable state: Significantly higher average levels of D-dimer, CRP as well as decreased fibrinogen levels were found in COVID-19 patients who had cerebrovascular manifestations reported by Li et al. [31], indicating hypercoagulable states resulting from inflammation and leading to stroke [32]. Based on this hypercoagulability, significant vessel stenosis was found in five of 11 patients (45.5%) in a study by Li et al. [31]. Large vessel occlusion and hemorrhagic transformation of an ischemic stroke with COVID-19 or more susceptible to intracerebral hemorrhage (ICH) [33]. 

Vasculitis and pathogenesis of ischemic stroke due to ACE-2 receptors: According to Varga et al., the vascular endothelium is rich in ACE-2 receptor that, when exposed to the COVID-19 virus, causes an inflammatory response (a lymphocytic “endotheliitis”) that both creates a surplus of immune cells and acts as one of the substrates for the thrombotic complications [34].

While the exact theory of how hemorrhagic stroke with COVID-19 infection occurs is still uncertain, some idea of the ACE-2 receptors on the brain endothelial and arterial smooth muscle cells allows the vulnerability and damage by the virus, enabling the vessel to be more prone to rupture [35]. Few case reports mention that the pathogenesis could be a multifactorial combination of small vessel vasculitis, microvessel thrombosis, dysfunction of the endothelial lining, or even imbalance of the RAAS system causing the risk for stroke [36]. The SARS-CoV-2 spike protein surface unit interacts by entering the cell membrane-bound protein with the aid of an angiotensin-converting enzyme (ACE-2) receptor, which is known to have the effects of causing vasodilation via its antihypertensive, antifibrotic and antiproliferative effects. This dysregulation, especially in endothelial cells, can lead to the inhibition of fibrinolysis and further thrombin production [37].

COVID-19 severely affected ICU patients who needed ECMO and were treated with higher doses of anticoagulants which could be a reason for getting more hemorrhagic stroke than ischemic stroke as a complication [38]. Pathogenesis may be related to SARS-CoV-2 with an affinity for ACE-2 receptors, allowing the virus to damage intracranial arteries causing vessel walls to rupture and cause hemorrhage [35]. Cytokine storms could also cause hemorrhagic strokes, as reported in a diagnosed CO­VID-19 patient who developed an acute necrotizing encephalopathy associated with late parenchymal brain hemorrhages [39].

Pathophysiology with interferons and cytokine storms: Analytics found IL-2, IL-6, tumor necrosis factor (TNF), and anti-phospholipid antibodies were profoundly activated by the human immune system, which then triggered the massive response to the “cytokine storm,” which degraded the extracellular matrix (ECM), the lining of the endothelial lumen. Plaques from ECM degradation and blood components of substances were the triggering factors for blood to be hypercoagulable and stagnant, a risk especially for ischemic and some hemorrhagic strokes [40]. However, IL-6 may also help increase post-stroke angiogenesis and provide protection in ischemic stroke [41]. Patients can have confusion and altered states of consciousness due to proinflammatory cytokines that increase markers hypercoagulation increasing the risk of stroke. Dodd et al.'s study analyzed the inflammatory response triggered by COVID-19 infection could be the factor to cause saccular premature aneurysms to rupture and subarachnoid hemorrhage [42].

Pathophysiology of PD and COVID-19: According to Brown et al. [9], 18% with PD also reported new motor symptoms and worsening previous symptoms during the pandemic. The possible mechanisms for aggravating symptoms are a systemic inflammatory response to the SARS-CoV-2 virus [43], social isolation [44], discontinuation or miss in medication doses, lack of exercise, social activities, and missing follow-up visits [45].

Pathophysiology of Alzheimer/dementia and COVID-19: Aggravation of Alzheimer’s disease symptoms related to increased expression of ACE-2 receptors [46]. Direct neurotoxicity by activation of host immune response. APOE Ꜫ4 expression, Aβ and tau protein deposition [47], and oxidative stress. Age, gender, hypertension, cardiovascular disease, diabetes mellitus, and pneumonia are common risk factors and comorbidities. Aging causes increased production of ROS, disruption of BBB, exacerbated Aβ production, and neuroinflammation, which causes pathogenesis of both Alzheimer’s disease and COVID-19 [48]. Also, ischemic white matter damage [49], Blood-Brain Barrier damage, cerebral hypoperfusion, which increases the phosphorylation of tau protein [50], and cerebral accumulation of Aβ amyloid is potentiated by COVID-19 and potentially favor Alzheimer’s disease development [51]. Short-term mechanical ventilation due to ARDS in COVID-19 causes Aβ peptide accumulation and Blood Brain Barrier dysfunction, potentiating Alzheimer’s disease development [52]. 

Pathophysiology of multiple sclerosis and COVID-19: Clinical relapse of MS is precipitated by auto-activation of peripheral T and B lymphocytes and resident CNS immune cells, which enter the CNS by crossing the blood-brain barrier and aggravating the demyelination of neurons [53]. Depletion of the antiviral CD8 response [54] results from the fact that in about 80% of COVID-19 patients, CD3 +ve, CD8 +ve, and CD4 +ve T cell counts are typically decreased, and recovery of the normal count of T lymphocytes takes place in between 2 and 12 months [55]. This may promote infection by latent microbes with the ability to trigger Multiple Sclerosis [56]. Additionally, alterations in intestinal pathogens and intestinal barrier breakdown can be detected as neuroinflammatory mediators by the gut-brain axis and may exacerbate MS by intensifying autoimmune responses. Fluctuations in the body or ambient temperature and febrile illnesses like COVID-19 can worsen neurological symptoms [57]. 

Pathophysiology of epilepsy and COVID-19: A proposed hypothesis is that the COVID-19 virus can enter the CNS via axonal transport, blood-brain barrier, or olfactory bulb [58]. Another exciting theory is that distress imposed by social isolation may lead to breakthrough seizures and aggravated neurological symptoms in patients with preexisting epilepsy. In addition, hypoxemia, drug-drug interactions, and metabolic abnormalities may also contribute to worsening neurological episodes in COVID-19 patients with epilepsy [8].

Neurological and non-neurological symptoms of COVID-19

Stroke

COVID-19 most commonly presents with fever, cough, sore throat, nausea, vomiting, anorexia, fatigue, and in severe cases, sepsis and pneumonia. SARS-CoV-2 has the propensity to affect both CNS and PNS. In the first 14 days in patients infected with SARS-CoV-2 infection, involvement of the peripheral and central nervous systems is well known [59]. Neurological symptoms during SARS-CoV-2 range from mild clinical features like anosmia, fatigue, dizziness, headache, and myalgia to severe clinical presentations like encephalopathy and ischemic strokes, hemorrhagic, and rare times aneurysms [60]. Difficulties in memory, poor concentration, visual disturbance, decreased consciousness levels, seizures, delirium, involuntary movements, and ataxia are also known [10]. About 20% of patients admitted to the ICU for COVID-19 reported neurological manifestations and complications, and those with neurological issues predating their COVID-19 diagnosis presented a higher mortality rate.

Stroke as the First Symptom of COVID-19

Several studies have contributed to our evolving understanding of the association between stroke and SARS-CoV-2 infection. In some cases, stroke was the presenting symptom for patients testing positive for COVID-19. In a multicenter study by Trifan et al. based in Chicago, 83 patients were included based on radiological evidence of stroke and positive COVID-19 diagnosis. Among them, 32 patients (39%) presented with stroke as the primary diagnosis. Ischemic Stroke was found to be the most frequent subtype (77%), intracranial hemorrhage (ICH) (19%) was second, followed by subarachnoid hemorrhage (SAH) (4%) [61]. The authors also sought to describe the association of stroke with COVID-19 in demographic distribution; for example, 53% were males among 83 patients included in the study. 75% were Hispanic or Black, and the median age was 64. They concluded that males had higher risks of getting severe COVID-19 infections and suffering from worse ischemic stroke outcomes than females [61]. The chances of a stroke are 7.6 times higher in COVID-19 patients than in patients with historic influenza infection. The young individuals with positive COVID-19 disorder who had no prior vascular risk factors got a large vessel stroke which is seven times higher, in contrast, another study demonstrated that irrespective of age, sex, and vascular risk, stroke was independently associated with COVID-19 [62]. Li et al. reported that among 221 patients, 13 (5.9%) patients were found to develop acute cerebrovascular events. Among them, 11 patients developed acute ischemic stroke, cerebral venous sinus thrombosis in one (0.5%), and ICH in one (0.5%) patient, respectively [31].

Symptoms of Stroke and Effects of COVID-19 on Pre-existing Stroke

Avula et al. elucidated that about four patients admitted in a medical emergency with acute stroke-like symptoms such as altered mental status, facial droop, slurred speech, left-sided weakness, right arm weakness, numbness and other symptoms [63]. COVID-19 patients with pre-existing cerebrovascular disease are at increased risk of developing poorer outcomes [64]. This is commonly seen in patients with at least one baseline vascular risk factor (VSF) in which arterial hypertension is the most prevalent. Those with stroke with VRF are likely to have a worse prognosis [65]. In a meta-analysis containing 47 included studies involving 7,267,055 patients, comorbid stroke was associated with an increased risk of mortality in Covid-19 patients (pooled effect = 1.30, 95% CI: 1.16-1.44, random-effects model) based on confounder-adjusted effect estimates [66]. Previous studies also suggest that patients with pre-existing cerebrovascular diseases might be prone to increased stroke risk with COVID-19 [33]. Even among non-infected patients, indirect consequences of the COVID-19 pandemic could increase stroke morbidity and mortality. Hypercoagulability and thromboembolism result from the robust inflammatory response caused by COVID-19 [67].

Extracorporeal Membrane Oxygenation (ECMO)

Although stroke has etiological causes like hypertension, atherosclerotic plaque formation, and dislodgement of emboli due to atrial fibrillation, other etiological factors also contributing to COVID-19 treatments causing a stroke is the use of ECMO or also known as extracorporeal life support. The importance of this technique is to prolong the consent to the cardio-respiratory system in those with an inadequate amount of gas exchange and lack of sustained perfusion due to circulatory collapse. Patients with severe states of COVID-19 with compromised pulmonary conditions due to terminal stages of pneumonia and low oxygenation, which failed to improve with oxygenation masks, started to show neurological instability and cardiac failure. These critical patients were then placed on ECMO to help strengthen their oxygenation. However, these patients on ECMO were unfortunately at risk of air embolism complications, which increased the risk of ischemic stroke and rarely intracranial hemorrhage due to embolism lodgement [68]. Patients on either veno-arterial (15%-18%) or venovenous (4%-13%) ECMO were at risk of neurological complications [69]. Based on a recent study by Cho et al., it is discovered that comparing the patient's status of those before being admitted into ICU without ECMO, the higher chance of occurrence of ischemic or hemorrhagic stroke occurred to those in ECMO during their ICU [38].

Robinson et al.'s study of 2,699 COVID-19-positive patients, the median age of 59, 65% male, 70% of patients were on mechanical ventilation, and 10.5% on ECMO. Of this cohort, 75 patients had strokes as a complication of hospital stay, and 16 were excluded for unknown stroke timing occurrence during their time of admission. Of these 59 patients studied out of the cohort, 27 (46%) patients had a hemorrhagic stroke, 19 (32%) patients had an ischemic stroke, and 13 (22%) patients were unspecified on their type of stroke based on the Imaging (CT or MRI) [70]. Hence, ECMO is shown to have a higher risk of hemorrhagic stroke than ischemic type. Robinson et al. compared ECMO patients developing stroke against those without ECMO developing stroke. In 283 patients with ECMO, 15 (5.3%) had a hemorrhagic stroke, three (1.1%) patients had an ischemic stroke, and four (1.4%) were unspecified strokes. Of these 283 patients, 94% (266) of the people had venovenous ECMO, and the remaining venoarterial ECMO supported 17 patients [70]. Those with stroke while being treated with ECMO were more critical patients with more frequent needs for mechanical ventilation and vasopressor treatment support. In the non-ECMO group, among 2,415 patients, 12 patients (0.4%) had a hemorrhagic stroke, 16 patients (0.6%) had an ischemic stroke, and nine patients (0.3%) were defined as having an unspecified type of stroke [70].

Hemorrhagic Stroke

Most patients from the general population who develop COVID-19-related stroke are commonly ischemic, although there have been a few hemorrhagic cases [71]. Trifan et al. [61] found the incidence of ICH to be 19% among his cohort of cases, 38% of which were discovered during hospitalization. The median Glasgow Coma Scale/Score (GSC) was 14, and the ICH score was 2 in clinically symptomatic patients (88%). ICH was to be most commonly caused spontaneously (44%) or was related to anticoagulation (44%) [61]. The mortality rate among the ICH patients with COVID-19 was 50%, with the modified Rankin Scale (MRS) of those who survived being 2 [61]. During hospitalization, many patients were placed on anticoagulation which increased the bleeding risk, particularly the cases of intracranial hemorrhage. Patients with oral anticoagulation had a higher mortality rate than those with a spontaneous cause of ICH [72].

Dogra et al. study that anticoagulation increases the risk of ICH [73]. In his study cohort of 3,824 COVID-19 positive patients, 33 patients were reported to have a hemorrhagic stroke: five (15.2%) patients with parenchymal hemorrhage resulting in herniation from mass effect with radiological imaging of diffuse hypoxic injury and mortality rate of 100% [73]. Females (21.2%); mean age of 61.6 years (range of 37-83 years) and mortality of 14 (42.4%) patients; 15 (45.5%) continued hospitalization and four (12.1%) patients discharged. Those with a high D-dimer were initiated with anticoagulants based on their hypercoagulability state. 2,204ng/mL was the median D-dimer before the hemorrhage was diagnosed. Anticoagulants used in the cohort were intravenous heparin, argatroban, or enoxaparin, of which 40mg was used subcutaneously daily or twice for those with BMI>40 and 30mg twice daily for BMI<40 [74]. Dogra et al.'s study of the remaining 28 (25%) patients who presented with punctate hemorrhage of the cortex, small hemorrhages were seen in 17 (60.7%) patients, and large single hemorrhages were found in four (14.3%) patients. Mortality was seen in eight (33.3%) patients with small and punctate hemorrhages and one (25%) in those with a single large hemorrhage without herniation [73].

Diagnostics Assessments and Predictors of Neurologic Manifestations for Stroke

For all patients suspected to present with stroke symptoms, apart from looking into their comorbidities and risk factors placing patients at higher mortality risk, the SARS-CoV-2 virus further exacerbated stroke to be the most prominent neurological adverse effect in those with positive COVID-19 infection. Evaluation of diagnosing parameters for stroke is the same in patients with or without COVID-19. Initiation of hypercoagulability testing profiles of activated partial prothrombin time (aPPT), prothrombin time (PT), d-dimer, and fibrinogen with complete blood count (CBC) to rule out hematologic aspects in mind. This too is evaluated at the time the patient's stroke lasts if <4.5hrs or >4.5hrs with the use of neurovascular and brain imaging to look for venous or arterial causes of cardiovascular reasons and whether the stroke is ischemic or hemorrhagic [75]. COVID-19 in few studies has shown that, unlike other viral infections, it can increase the chance of having an ischemic stroke to 7.6% with very high levels of D-dimers, which reflects the high clotting turnover and being an aggressive thrombotic, further increasing the chances of ischemic stroke. Furthermore, COVID-19 patients from the underlying hypercoagulable may also be at higher risk for a re-occlusion after thrombectomy done mechanically [76].

Eskandar et al. highlight the predators of patients presenting with neurological findings [12]. They found that patients who have a prior stroke history had comorbidities of renal disease (blood urea nitrogen >30 mg/dL, creatinine >1.5 µmol/L), heart failure, and chronic obstructive pulmonary disease (COPD) of black race or Latino ethnicity were more at risk. Looking at the parameters, he demonstrated those with hypercoagulability states of d-dimers >3 mg/l, <150,000/mm^3^ platelets, >1.2 international normalization ratio (INR) and >0.1 ng/mL procalcitonin and troponin >0.1 ng/ml were at higher risk as potential correlation to neurologic presentations [12]. 

Stroke Imaging Findings in COVID-19 Patients

Physical signs of stroke within patients, such as muscle paralysis and facial drooping, shortly after being infected with the SARS-CoV-2 may be considered surrogate markers of a systemic neurological disorder affecting the circulatory system. Diagnosis and confirmation of stroke are usually made with computed tomography (CT), and findings within the neuroimaging often consist of either acute infarction or hemorrhage. Ladopoulos et al. conducted a review of neurological disorders and their alternate manifestations of ischemic and hemorrhagic stroke within neuroimaging; summarizing that within COVID-19 patients, large vessel occlusion (LVO) was the most common observation in the total acute ischemic stroke (21.6%-72.5%), followed by multiple LVOs (14.9%) and lacunar strokes (7.5%-8.7%) with occasional hemorrhagic transformations of ischemic strokes [61]. The most common types of intracranial hemorrhage (ICH) include subarachnoid and intraparenchymal ICH of non-hypertensive locations of lobar, cortical, and junctions of cortico-subcortical areas. He also highlighted that critically ill COVID-19 patients on mechanical ventilation were prone to having multiple cerebral microbleeds (MCB) found in juxtacortical white matter. Trifan et al. showed the characteristics and neuroimaging evidence of acute ischemic stroke among 77% of patients whose most common infarct location was cortical. However, a quarter of patients had a bilateral hemispheric presentation [77]. Stroke of undetermined etiology (39%) was the most common, followed by cardio-embolism (27%). Treatment with tPA was done in 8%, and endovascular thrombectomy was done in 11%. The mortality rate among ischemic stroke patients with COVID-19 was 27%, and the modified Rankin Scale (MRS) at discharge was 4.

Implications of Therapy

Managing treatment during the pandemic was challenging due to the virus’s high morbidity and relative recency of emergence. Joint stroke management includes the use of anticoagulants, anti-inflammatory, and thrombolytics; however, with more knowledge of the SARS-CoV-2 affinity for ACE-2 receptors within endothelial lumens, the use of angiotensin receptor blockers (ARBs) and angiotensin-converting enzyme (ACE) inhibitors was started as a trial to help stabilize patients' symptoms [36]. Tang et al. reported that COVID-19 patients with coagulopathy could reduce mortality with anticoagulation treatment such as direct oral anticoagulants (DOAC), heparin, or warfarin [78]. According to Asakura and Ogawa, COVID-19-associated coagulopathy presents some features that may suggest disseminated intravascular coagulation (DIC) and recommended a combination of heparin and Nafamostat mesylate [79]. Anti-inflammatory therapeutics such as IL-6R monoclonal antibodies (tocilizumab), TNF-α inhibitors (etanercept and infliximab), and IL-1β antagonists (the monoclonal antibody canakinumab and the anti-cytokine anakinra) could potentially serve as effective COVID-19 management, based on their benefits in cardiovascular clinical trials [80]. However, immunosuppression, including corticosteroids, may hamper the elimination of the virus, and those with an impaired immune system have more risk of developing secondary infections [81]. As for antiviral therapy, Beigel et al. reported that in a randomized controlled trial in 1,062 patients hospitalized for COVID-19, Remdesivir was associated with a shorter recovery time compared with placebo (10 days in comparison to 15 days, 95% CI: 9-11 to 13-18), and lower 14-day mortality of 6.7% with Remdesivir versus the placebo’s 11.9% (hazard ratio for death, 0.73; 95% CI: 0.52-1.03) [82].

COVID-19 patients with stroke had higher chances of worse outcomes and more significant mortality than those without [83]. Patients with a history of CNS involvement had the worst prognosis. Due to the pandemic, utilization and access to healthcare were interrupted, which may have deterred stroke patients from seeking appropriate care, falsely affecting data. The mortality rate was seen to be high in most COVID-19 patients after stroke manifestations, with further highlighting factors of elevated d-dimers reflecting a hypercoagulable state from the effects of the viral infection’s impact on the endothelial cell. 

Parkinson's Disease

PD is a neurodegenerative disorder commonly presenting with motor and non-motor symptoms [84]. Worsening of both motor and non-motor symptoms of PD during COVID-19 infection has been reported in previous literature (Table 2). A systematic review said that 60% of patients with pre-existing Parkinson's or dementia developed worsening neurological symptoms [19]. Among the reported worsened symptoms, bradykinesia was the most common one. Other motor symptoms were gait disturbances, tremors, and rigidity. Aggravated non-motor symptoms were-sleep disturbances, cognition, autonomic, mood disorders like anxiety, depression, appetite disorders, repeated falls, and pain [9]. Some studies also looked at the new diagnosis of Parkinson's following COVID-19 infection. Li et al. reviewed a temporal relationship between new-onset parkinsonism and COVID-19 disorder. These cases had no obvious motor symptoms or positive family history before getting infected by SARS-CoV-2 [9]. However, we could not exclude the possibility of the early stage of undiagnosed PD [85].

Another intriguing link to consider in PD patients is the severity of COVID-19 symptoms in this population. One systematic review showed the hospitalization rate among PD patients along with COVID-19 was about 40%, and the mortality rate was about 25% [11]. Based on our study, we could say that some of the following factors may play an essential role in developing severe COVID-19 in Parkinson's patients with increased hospitalization or ICU admission. People with Parkinson's disease with COVID-19 seem to have an increased risk of pneumonia and clinical deterioration that led to hospitalization [86]. According to Sulzer et al., the commonest cause of hospitalization is a severe infection due to high viral load or pro-inflammatory conditions that increase the need for supported breathing and mechanical ventilation in older PD patients [87]. Another factor to consider is older age and male sex [88]. The average age of PD diagnosis is around 60±10 years for male patients. Older PD patients tend to present with some atypical symptoms of COVID-19, such as delirium and functional impairment without obvious physical symptoms [88]. Furthermore, complex therapeutic management and limitations in in-patient consultation led to more hospitalization. From all the published reports, we found that the duration of PD is also an essential factor [89]. We can say the more extended the PD duration, the higher the risk for poor outcomes from COVID-19 [90]. Patients with long-term Parkinson's suffer more severe illnesses requiring hospitalization and ICU admission. Other pre-existing comorbidities in PD patients, such as hypertension, heart disease, and diabetes, seem linked to severe COVID-19 infections [88]. Based on these previous studies, we can say that PD significantly affects the severity and prognosis of COVID-19 conditions.

Delirium

Delirium was identified when an electronic medical record (EMR) described the COVID-19-affected patient as being altered or confused. The new confusion is now a CDC emergency warning sign for COVID-19, and the results of this cohort suggest that about one-third of people with dementia present with delirium [16]. Patients who were previously diagnosed with dementia are more likely to develop delirium (36.2% vs. 11.6%, p < 0.001), and a post-doc logistic regression showed that this remains significant after age and gender adjustment. These patients were also less likely to exhibit some other COVID-19 symptoms on presentation [16]. According to a European study, people who had dementia and were later affected by COVID-19 may be more likely to experience delirium, considered as an independent risk factor in the cases of increased in-hospital mortality [91]. A previous study with Italy's first COVID-19 wave confirmed that delirium was the first sign in 36.8% of patients with dementia, which has shown high mortality in these patients [92]. In another Italian cohort, delirium associated with COVID-19 has been associated with increased mortality but is not an independent risk factor [93].

Alzheimer’s Disease/Dementia

Alzheimer’s disease represents the most common form of dementia worldwide [89]. Patients with Alzheimer's disease are at increased risk of contracting SARS-CoV-2 and show an increased mortality rate [23]. On the other hand, COVID-19-affected patients seem to be more prone to Alzheimer’s disease. After penetrating the brain, the virus can cause demyelination, neurodegeneration, and cellular senescence, which may accelerate the development of Alzheimer's disease [94]. As Alzheimer's patients depend on caregivers and physiotherapists for exercise, isolation and confinement during COVID-19 may exacerbate their symptoms [89]. One study mentioned that more than 30% of hospitalized COVID-19 patients exhibit neurological manifestations [95]. In a postmortem brain MRI of 62 patients who died of COVID-19, four MRIs showed brain structural changes associated with COVID-19 [96]. A study demonstrated that biomarkers for neurodegeneration such as neurofilament light chain protein (NFL), tau proteins, and glial fibrillary acidic protein (GFAP) are increased in the cerebrospinal fluid of COVID-19 patients by 63%, 37%, and 16%, respectively [97]. Convelli et al. conducted a study to find the connection between COVID-19 and dementia and found that worsening symptoms of dementia were present in nearly one-third of the sample. In particular, memory and orientation abilities declined the most. Among the examples, 13.6% developed a reduced level of independence and functional decline [98].

Multiple Sclerosis

A chronic inflammatory illness that affects the central nervous system, multiple sclerosis is characterized by varying degrees of neurodegeneration brought on by localized demyelination [99]. In an observational study at NYU Langone, the authors (Parrottaa et al. reported confirmed or suspected COVID-19 patients with MS. Among 76 patients, 55 had relapsing MS, 17 had progressive MS, and four had a related disorder [24]. Fever and cough were the most common symptoms (Table 1), with 21.1% diagnosed with neurologic symptom relapse preceding or coinciding with infection. Among these patients, 23.7% were hospitalized, and 10.5% had critical illness or death. Most patients, despite being on DMT, did not require hospitalization. Most hospitalized patients were older, had progressive MS subtype, needed ambulatory assistance, or were non-ambulatory and had comorbid obesity. 10.5% of patients with a critical illness were more aged, had progressive subtype, and required assistance for ambulation/ non-ambulatory. High rates of comorbid obesity (62.5%), venous thromboembolism (37.5%), and CAD (25%) were found in the critically ill group. One patient died due to hypercoagulability resulting in multiple venous thromboembolism.

In another longitudinal cohort study in the UK, Garjani et al. demonstrated that 30% and 12% of non-hospitalized COVID-19 patients with MS experienced prolonged COVID-19 symptoms for ≥4 and ≥12 weeks, respectively [15]. These symptoms are higher than the general population, as reported by Sudre et al. [100]. COVID-19 may be prevalent for a longer time in MS patients as COVID-19 causes MS exacerbations as it shares neurologic symptoms of MS [15]. More than 80% of COVID-19 patients with MS showed additional signs not typical of MS. Pre- COVID-19 neurologic disabilities make patients susceptible to long-term sequelae of COVID-19, as reported by Salter et al. [101]. Pre-COVID-19 mental health issues predispose patients to PASC [102]. The limitation of these studies is that the MS population is only a proportion of participants.

The American Academy of Neuromuscular and Electrodiagnostic Medicine and the National Multiple Sclerosis Society both emphasized the significance of vaccinating MS patients receiving immunotherapies against SARS-CoV-2, irrespective of their immunosuppressive status. Vaccination should not be delayed, and patients should be educated to remain observant even after vaccination due to the possibility of absent or depleted immunity against the virus, as immunotherapy can reduce vaccine response [103]. To determine vaccine effectiveness, SARS-CoV-2 antibody levels can be assessed after vaccination [104]. Additionally, DMT-treated patients had a higher likelihood of contracting SARS-CoV-2 [13]. Disease-modifying therapies (DMTs) might increase the chance of developing complications from a COVID-19 infection, too, so the risk-benefit ratio should be measured before stopping treatment, as suggested by The Multiple Sclerosis International Federation [21].

Epilepsy

Epilepsy is a significant component of pre-existing neurological triggers. In a study conducted among hospitalized patients with COVID-19 in Mexico City, 15 patients previously who had epilepsy developed worsening neurological symptoms [14]. Headache and myalgia were the most frequently reported symptoms in the patients (Table 1), standing at 41.7% and 38.5%, respectively. Other common complaints were delirium (13.1%), parageusia (8%), anosmia (7%), limb weakness (5.1%), and distorted mental status after withdrawal of sedating medications (2.5%) [14]. This was further strengthened by two studies conducted where the seizure was reported with increased frequency in COVID-19 with a previous history of epilepsy [40,105]. Furthermore, the mortality risk was twice as high in COVID-19 patients with a background of epilepsy [102]. Still, enough data was not found to conclude that it was an independent factor determining mortality. According to some societies, the risk of sudden unexpected death in epilepsy may be increased due to COVID-19. According to some research, sudden unexpected death in epilepsy (SUDEP) may be more likely due to infection or viral disease. However, there is still no evidence that COVID-19 and SUDEP are linked.

Interestingly, COVID-19 itself may lead to the development and worsening of epilepsy [106]. In 2020 in the UK, people mentioned a change in seizures (19%), mental health difficulties (34%), and sleep disruption (26%). A survey has been done on patients with epilepsy (68%) and their caregivers (32%) [107]. Anxiety, depression, social isolation, physical inactivity, and disruptions to social life are all examples of stressors that can increase seizure frequency. Other factors such as decreased adherence to anti-epileptic medication and difficulty in acquiring access to healthcare can contribute to increased epileptic attacks [108]. Statistically significant data were reported in which 13% demonstrated impaired adherence to medication due to changes in routine (8%), problems in acquiring prescriptions (3%), and stress (7%). According to reports, many epilepsy clinics closed their doors during the pandemic. There was also a significant delay in imaging and intervention for epilepsy [107]. This led to vulnerable populations, including Black, Asian, and Minority Ethnic (BAME) groups, the elderly, and people from lower socioeconomic incomes, being more affected due to health service inequality [107].

## Conclusions

COVID-19 has led to developing and worsening multiple neurological disorders, including stroke, Parkinson’s disease, multiple sclerosis, epilepsy, Alzheimer’s disease, and delirium. On the other hand, the pre-existing neurological conditions triggered increased severity during COVID-19, such as the increased requirement for ICU management, and those with pre-existing dementia led to the development of delirium. The medications used to treat pre-existing neurological disorders may play a significant role in developing complications. Furthermore, specific social barriers such as social isolation and difficulty acquiring medications may have triggered an increased frequency of epileptic episodes. In addition to the development and worsening of symptoms, it has also led to increased risk for hospitalization and mortality. We believe that this systematic review has shed important light on the relationship between neurological disorders and COVID-19. It will encourage medical personnel to take extra caution while managing neurological impairments, such as monitoring for stroke using ECMO, improving access to anti-epileptic medications, and considering the risk-benefit ratio of using disease-modifying drugs in multiple sclerosis. Further research is needed to understand this phenomenon, and this systematic review has significantly contributed to the existing literature.
